# Cerumenolytic Effects of Carbamide Peroxide in Patients with Ear Wax Obstruction

**DOI:** 10.22038/IJORL.2024.67777.3311

**Published:** 2024-03

**Authors:** Alireza Asgari, Hamid Reza Asgari, Mehrdad Ghorbanlou, Faramarz Dobakhti, Mohammad Ali Ghorbanian

**Affiliations:** 1 *Faculty of Pharmacy, Zanjan University of Medical Sciences, Zanjan, Iran.*; 2 *Department of Anatomy, School of Medicine, Iran University of Medical Sciences, Tehran, Iran.*; 3 *Department of Otolaryngology, Valiasr Hospital, School of Medicine, Zanjan University of Medical Sciences, Zanjan, Iran* *.*

**Keywords:** Carbamide peroxide, Phenol glycerin, Cerumen compaction, Ear

## Abstract

**Introduction:**

Accumulated and compacted ear wax or cerumen can cause conductive hearing loss, discomfort and vertigo, and infection. This study investigates the effect of Carbamide peroxide (CP) compared with Phenol glycerin (PG) ear drops on cerumen.

**Materials and Methods:**

This experimental study investigated the effect of PG and CP ear drops on cerumen in ex vivo and in vivo phases. In the ex vivo phase cerumen degredation was scored following PG and CP treatments. In the in vivo phase, 29 patients with bilateral cerumen impaction were randomly entered the study. PG and CP were applied 3 times a day (each time 5 drops) for 4 days by patients. After treatments, the time of cerumen removal was measured.

**Results:**

Instant changes showing degredation of cerumen (grade 1) was evident when it was exposed to CP, on the other hand degredation changes (grade 1) in cerumen treated with PG was only evident after 20 min incubation at 37 ^o^C, while grade 3 degredation was evident in cerumen treated with CP after the same time incubation. Although the time needed for removal of cerumen was lower in CP treatment (54.10±31.77) compared to PG treatment (67.10±35.54), the difference was not statistically significant.

**Conclusion:**

Based on the literature and our results, carbamide peroxide is suggested as a proper treatment for patients with EAC obstruction caused by cerumen compaction, because not only it is significantly effective in cerumen degredation, but also no side effects have been reported.

## Introduction

As a normal bodily secretion, cerumen or earwax, lubricates, protects, and cleans the external auditory canal (1,2). Ear wax is produced by secretion of apocrine sweat glands combined with sebaceous glands resulting in a secretion composed of fatty acids, cholesterol, ceramides, wax esters, long chain hydrocarbons and etc (3). It is believed cerumen is eliminated or removed naturally by epithelial migration and the help of temporomandibular joint action (2, 4). When its natural removal is disturbed, wax accumulation and subsequent compaction will be resulted; among the causes of wax accumulation, self-cleaning or putting things in the ear such as headphones and hearing aids are the common ones (1). The consequences of wax accumulation can be conductive hearing loss, discomfort and vertigo, and infection (5). Men, elderly and intellectually impaired individuals are more prone to wax obstruction (6,7). 

Ten percent of children and five percent of the adult population are involved in this condition (2,8). The two types of ear wax show an ethnicity/race correlation in which the dry type is dominantly seen in East Asians (95%), but rarely in Europeans and Africans (3%), and the mixed type is more commonly seen in North Americans, Central Asia, and Turkey (30-50%) (3,9). Several approaches and medications are suggested in the literature for wax removal including mechanical methods which includes dry method with the aid of microscope and ear curette, and wet method by syringing with body temperature water (3). 

To avoid these interventions or assist the removal procedure, there are several medication in the form of drops which help in disintegration of wax – cerumenolytics (1,3). These medications include oil-based compounds (such as olive oil), water-based compounds (such as sodium bicarbonate), a combination of oil and water-based compounds, and non-water, non-oil solutions (such as carbamide peroxide and glycerol). In this study, we aimed to investigate the cerumenolytic effect of Phenol Glycerol and Carbamide Peroxide eardrops in an in vivo and ex vivo approach in patients with various degrees of wax obstruction. 

## Materials and Methods

 In this experimental study which was carried out in ENT department of Valiasr hospital and pharmacology department of Zanjan University of medical sciences, two study phases were included: ex vivo and in vivo. 

*Ex vivo study: *Cerumen from 30 different patients who presented with hearing loss symptoms, without age and sex discrimination was collected by an otorhinolaryngologist, by obtaining informed consent. 

Then the combination of the cerumen was divided into 30 tubes each containing 200 mg cerumen. Fifteen tubes containing cerumen were exposed to 10 ml of Phenol Glycerol 6.4% ear drop (containing 6.4 gr phenol per 100 gr, 10 ml ear drop, Sepidaj pharmaceutical Co., Tehran, Iran) and the cerumen in the other fifteen tubes were exposed to 10 ml of Carbamide Peroxide 6.5% (made by dissolving 6.5 gr hydrogen peroxide-urea in 100 ml of glycerin). 

Tubes were incubated while shaking for 20 minutes in 37 C^o^ and 46 rpm. After that, samples were observed and scored (0: no change, 1: little degredation, 2: moderate degredation, 3: much degredation, 4: complete degredation). Scoring was done according to the changes of the appearance including inflation, and degredation. Observation of the samples were done several times including four observations of the incubated samples with twenty minute intervals and then following 24, 48, and 72 hr. 

*In vivo study: *For in vivo investigations, 29 patients aging 9 to 78 years old (male=12, female=17) with bilateral cerumen impaction were randomly entered the study. 

The amount of external auditory canal (EAC) obstruction was scored as follows: 0 (no obstruction), 1 (obstruction ≤25% of the EAC diameter), 2 (25% < obstruction ≤ 50% of the EAC diameter), 3 (50% < obstruction ≤ 75% obstruction of the EAC diameter), 4 (75% < obstruction ≤ 100% of the EAC diameter). PG and CP were applied 3 times a day (each time 5 drops) for 4 days by patients. In order to investigate the effect of PG and CP in reasonably similar conditions, they were applied on the left and right ear of the same patient, respectively, to avoid the influence of structural and genetic differences of cerumen. 

After 4 days, patients referred to the otorhinolaryngologist for removal of ear wax. Then the time for removal of cerumen was recorded and compared between the two medications. 

*Statistical analysis: *Degree of obstruction between the left and the right EAC was compared by independent t-test which showed no significant difference indicating the reliability of the data provided. The time of cerumen removal was also compared by independent t-test between the two medications. Linear regression was performed to reveal the correlation between left and right EAC obstruction, and EAC obstruction and time of removal. Data were reported in mean ± standard deviation (SD), and P ≤ 0.05 was considered significant. 

## Results

*Ex vivo effects of Phenol Glycerol and Carbamide Peroxide*

**Fig 1:**
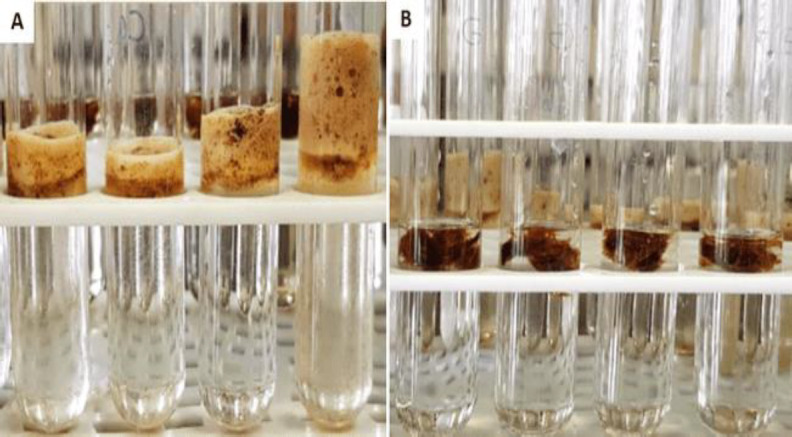
Degradation of cerumen exposed to Phenol Glycerol (PG) and Carbamide Peroxide. (A): CP-treated cerumen after 20 min incubation (grade 3 degradation); (B): PG-treated cerumen after 20 min incubation (grade 1 degradation)

Instant changes showing degredation of cerumen (grade 1) was evident when it was exposed to CP, on the other hand degredation changes (grade 1) in cerumen treated with PG was only evident after 20 min incubation at 37 ^o^C, while grade 3 degredation was evident in cerumen treated with CP after the same time incubation. After 40 min incubation, CP-treated cerumen degraded to grade 4, but PG-treated cerumen degraded to grade 2. Prolonged incubation for 72 hr showed degredation of grade 3 in PG-treated cerumen (Figure 1). 

*In vivo effects of Phenol Glycerol and Carbamide Peroxide:*

Since PG and CP were applied on left and right EAC of a same patient, respectively, obstruction degree of right and left ears were investigated which showed no significant difference (left EAC= 85.25±23.5, and right EAC: 86±21.5) (Table.1).

**Table 1:** Demographic data, EAC obstruction degree, and time of cerumen removal.

**Patient**	**Left EAC obstruction degree (%)**	**Wax removal time (PG treatment) (s)**	**Right EAC obstruction degree (%)**	**Wax removal time (CP treatment) (s)**	**Gender**	**Age**
1.	4 (100)	75	4 (100)	50	M	67
2.	3 (75)	75	2 (50)	30	M	39
3.	4 (100)	60	3 (75)	10	M	28
4.	2 (50)	30	2 (50)	20	M	12
5.	2 (50)	70	2 (50)	30	F	23
6.	4 (100)	130	4 (100)	140	F	65
7.	2 (50)	60	2 (50)	60	F	38
8.	4 (100)	30	4 (100)	45	M	34
9.	4 (100)	90	2 (50)	70	F	24
10.	4 (100)	120	4 (100)	70	M	75
11.	4 (100)	70	4 (100)	90	F	29
12.	1 (25)	20	3 (75)	50	F	46
13.	2 (50)	70	4 (100)	50	F	47
14.	4 (100)	30	4 (100)	30	M	45
15.	4 (100)	50	4 (100)	90	F	45
16.	2 (50)	30	4 (100)	30	F	50
17.	4 (100)	50	4 (100)	30	F	73
18.	4 (100)	75	4 (100)	30	F	40
19.	4 (100)	70	4 (100)	45	F	40
20.	4 (100)	90	4 (100)	105	M	78
21.	4 (100)	30	2 (50)	30	F	59
22.	4 (100)	120	4 (100)	60	M	9
23.	4 (100)	50	4 (100)	70	F	47
24.	4 (100)	150	4 (100)	120	M	28
25.	3 (75)	20	2 (50)	20	M	64
26.	4 (100)	90	4 (100)	70	F	32
27.	4 (100)	120	4 (100)	70	M	18
28.	4 (100)	30	4 (100)	30	F	44
29.	2 (50)	41	4 (100)	24	F	58
Total	3.41±0.94 (85.25±23.5%)	67.10±35.54	3.44±0.86 (86±21.5%)	54.10±31.77	M: 12	43.34±18.55
					F: 17	

Obstruction degree [1 (25%), 2 (50%), 3 (75%), 4 (100%)]

Linear regression results indicated that there is a significant correlation between left and right 

EAC obstruction (r=0.42, P=0.02) (Table.2). 

**Table 2:** Linear regression results for correlation of left and right EAC obstruction (considering left EAC obstruction as a dependent variable)

**Dependent variable**	**r**	**B**	**r** ^2^	**p-value**	**Formula**
Left EAC obstruction	0.42	0.45	0.17	0.02^*^	1.84 + 0.45 (right EAC obstruction degree)

*significant correlation between left and right EAC obstruction. Abbreviations: r – Pearson’s correlation coefficient; B – Model coefficient; r^2^ – square of Pearson’s correlation coefficient; formula predicts the left EAC obstruction based on the degree of right EAC obstruction.

 Although the time needed for removal of cerumen was lower in CP-treated EAC (54.10±31.77) compared to PG-treated EAC (67.10±35.54), the difference was not statistically significant (Table.1). Linear regression analysis indicated the significant correlation of left (PG treatment) and right (CP treatment) EAC obstruction degree and cerumen removal time, respectively (r=0.40, P=0.02; r=0.37, P=0.05) (Table.3). 

**Table 3:** Linear regression results for correlation between EAC obstruction (left (treated with PG) and right (treated with CP)) and cerumen removal time (considering cerumen removal time as a dependent variable)

**Dependent variable**	**Independent variable**				
Cerumen removal time of left EAC	Left EAC obstruction (treated with PG)				
	r	B	r^2^	p-value	Formula
	0.40	15.32	0.16	0.02*	14.77 + 15.32 (left EAC obstruction degree)
Cerumen removal time of right EAC	Right EAC obstruction (treated with CP)				
	r	B	r^2^	p-value	Formula
	0.37	13.49	0.13	0.05*	7.58 + 13.49 (right EAC obstruction degree)

*significant correlation between cerumen removal time and EAC obstruction. Abbreviations: r – Pearson’s correlation coefficient; B – Model coefficient; r^2^ – square of Pearson’s correlation coefficient; PG (Phenol Glycerol); CP (Carbamide Peroxide); formula predicts the cerumen removal time based on the degree of EAC obstruction.

## Discussion

In this study the effect of two ear drops on the degrading changes and removal time of cerumen were investigated in ex vivo and in vivo approaches. Early significant changes of the cerumen were observed when it was exposed to CP, and following 40 min incubation, complete degredation was evident. On the other hand, PG-treated cerumen were not significantly affected after 20 min incubation, and following 72 hr treatment, degredation grade reached to 3. 

Cerumen removal time after treatment with CP was decreased compared to PG treatment, but it was not significant. Significant correlation between left and right EAC obstruction was also observed. Also, cerumen removal time was significantly correlated with EAC obstruction. 

As the most common procedure performed in an ear, nose and throat (ENT) center, cerumen removal is of significant importance (8). 

Since cerumen removal usually occurs spontaneously, it was reported some of the patients with EAC obstruction were healed without any treatments (1). Several studies investigated the effect of ear drops on cerumen including Carr *et al.*, showed the cerumenolytic effects of aqueous sodium bicarbonate and aqueous acetic acid in children (10); Dummer *et al.*, reported the positive effects of two cerumenolytics – Audax (choline salicylate and polyoxypropylene glycol) and Cerumol (arachis oil) – in cerumen softening and gaining satisfaction of patients and specialists (11); Jaffe *et al.*, investigated the effect of Otocerol (phenazone and sodium carbonate) and Cerumol on the cerumen, and reported better results in Otocerol-treated patients such as less syringing and easier syringing, less pain and irritation (12); Lyndon *et al,*. compared the effect of Audax and Earex (arachis oil, almond oil, and rectified camphor oil) on cerumen, and reported Audax as a more efficient medication with less side effects, and less cerumen impaction post-treatment (13). Table 4 demonstrates various studies investigating the effect of different ear drops on cerumen. 

**Table 4:** Literature review on ear drops

**Study**	**Participants**	**Intervention**	**Outcomes**
**Carr et al. (** 10 **)**	**67**	**10% aqueous sodium bicarbonate**	**Extent of wax clearance by change in score for cerumen**
Dummer et al. (11)	50	choline salicylate and polyoxypropylene glycol condensate (Audax)	Amount of wax
Jaffe et al. (12)	106	phenazone and sodium carbonate (Otocerol)	Degree of wax clearance
Keane et al. (5)	97	arachis oil 57.3%, chlorobutanol 5%, paradichlorobenzene 2%) (Cerumol)	Proportion of patients with complete clearance of wax
Lyndon et al. (13)	35	choline salicylate 20%, ethylene oxide-polyoxypropylene glycol, glycol and glycerol (Audax)	Proportion of participants with complete clearance of wax
Meehan et al. (14)	48	docusate sodium (Colace)	Extent of wax clearance
Oron et al. (15)	38	carbamide peroxide and anhydrous glycerin (Auro), chlorobutanol, arachis oil and dichlorobenzene (Cerumol), and squalane, spiramint oil and paraffin (CleanEars)	Extent of wax clearance
Singer et al. (16)	50	docusate sodium (Colace), and triethanolamine polypeptide (Cerumenex)	Proportion of patients with complete clearance of wax
Vanlierde et al. (17)	40	almond oil	Reduction in wax from grade 3 or 4 to grade 2
Whatley et al. (18)	92	docusate sodium (Colace), triethanolamine polypeptide (Ceruminex), and saline	Proportion of ears with complete visualization of tympanic membrane

 When regarding the extent of wax clearance, there was no significant difference between oil-based ear drops and carbamide peroxide. However, side effects such as itchiness and bad smell were reported in oil-based ear drops, but studies regarding carbamide peroxide did not report any such side effects (15). 

## Conclusion

 Based on the literature and our results, carbamide peroxide is suggested as a proper treatment for patients with EAC obstruction caused by cerumen compaction, because not only it is significantly effective in cerumen degredation, but also no side effects such as itching, bad smell, and otitis externa have been reported. 
